# Aldosterone Induces Renal Fibrosis and Inflammatory M1-Macrophage Subtype via Mineralocorticoid Receptor in Rats

**DOI:** 10.1371/journal.pone.0145946

**Published:** 2016-01-05

**Authors:** Beatriz Martín-Fernández, Alfonso Rubio-Navarro, Isabel Cortegano, Sandra Ballesteros, Mario Alía, Pablo Cannata-Ortiz, Elena Olivares-Álvaro, Jesús Egido, Belén de Andrés, María Luisa Gaspar, Natalia de las Heras, Vicente Lahera, Juan Antonio Moreno

**Affiliations:** 1 Department of Physiology, Faculty of Medicine, Complutense University, 28040, Madrid, Spain; 2 Renal, Vascular and Diabetes Research Lab, IIS-Fundación Jiménez Díaz, Autonoma University (UAM), 28040, Madrid, Spain; 3 Department of Immunology, Centro Nacional de Microbiología, Instituto de Salud Carlos III (ISCIII), Majadahonda, 28220, Madrid, Spain; 4 Department of Pathology, IIS-Fundación Jiménez Díaz, Autonoma University (UAM), 28040, Madrid, Spain; National Cancer Institute, UNITED STATES

## Abstract

We aimed to evaluate macrophages heterogeneity and structural, functional and inflammatory alterations in rat kidney by aldosterone + salt administration. The effects of treatment with spironolactone on above parameters were also analyzed. Male Wistar rats received aldosterone (1 mgkg^-1^d^-1^) + 1% NaCl for 3 weeks. Half of the animals were treated with spironolactone (200 mg kg^-1^d^-1^). Systolic and diastolic blood pressures were elevated (p<0.05) in aldosterone + salt–treated rats. Relative kidney weight, collagen content, fibronectin, macrophage infiltrate, CTGF, Col I, MMP2, TNF-α, CD68, Arg2, and SGK-1 were increased (p<0.05) in aldosterone + salt–treated rats, being reduced by spironolactone (p<0.05). Increased iNOS and IFN-γ mRNA gene expression (M1 macrophage markers) was observed in aldosterone + salt rats, whereas no significant differences were observed in IL-10 and gene ArgI mRNA expression or ED2 protein content (M2 macrophage markers). All the observed changes were blocked with spironolactone treatment. Macrophage depletion with liposomal clodronate reduced macrophage influx and inflammatory M1 markers (INF-γ or iNOS), whereas interstitial fibrosis was only partially reduced after this intervention, in aldosterone plus salt-treated rats. In conclusion, aldosterone + salt administration mediates inflammatory M1 macrophage phenotype and increased fibrosis throughout mineralocorticoid receptors activation.

## Introduction

Previous studies have demonstrated the importance of aldosterone in inflammatory and fibrotic processes development when related to kidney diseases [1]. The effect of aldosterone on salt and water homeostasis and potassium excretion has been considered as its main renal effect. However, it has been shown that aldosterone plays an important role in the progression of renal disease not only because of hemodynamic effects [2]. Aldosterone stimulates sodium reabsorption in the kidney leading to elevation of blood pressure and hypertension. In addition to hypertension, a number of studies have reported that chronic administration of aldosterone in the setting of salt intake causes glomerulosclerosis and interstitial renal fibrosis [3] via its mineralocorticoid receptors (MR) promoting renovascular hypertrophy [4–6]. The aldosterone-effects are mediated by the MRs and clinical studies have confirmed the beneficial effect of MR antagonism on renal disease [7]. Data from experimental models have confirmed that the beneficial effects of MR antagonism are related to inhibition of aldosterone-mediated pro-inflammatory and pro-fibrotic effects [8,9].

Recently, it has been recognized the importance of heterogeneity of macrophage polarization in the feature of renal disease [10]. Classically activated macrophages, also called M1 macrophages, are activated by pro-inflammatory cytokines, resulting in their potent microbicide functions that also contribute to tissue inflammation, fibrosis and damage. Macrophages can be alternatively activated to the M2 phenotype which are involved in tissue remodeling [11]. The macrophage plays a key role in renal inflammation, fibrosis and remodeling induced by aldosterone and high salt intake. It has been shown, that aldosterone-mediated fibrosis is preceded by macrophage infiltration and increased expression of inflammatory markers in the kidney [9]. In cultured macrophages, aldosterone induces classical macrophage activation to the M1 pro-inflammatory phenotype, increasing production of pro-inflammatory cytokines such as tumor necrosis factor alpha (TNF-α), chemokine (C-C motif) ligand 2 (CCL2) and CCL5 promoting the release of pro-fibrotic proteins; transforming growth factor beta (TGF-β) and plasminogen activator inhibitor-1 (PAI-1). The pro-inflammatory and pro-fibrotic effects of aldosterone are prevented by MR antagonism or MR deletion in macrophages [12,13]. However, the specific role of aldosterone on macrophage differentiation *in vivo* has not been fully addressed.

We aimed to evaluate whether aldosterone+salt administration induces M1 polarization in rat kidney and whether MR blockade prevent this pro-inflammatory macrophage subtype. Structural, functional and inflammatory renal alterations produced by aldosterone+salt administration were also studied. Treatment with spironolactone, a MR antagonist [14], was evaluated to prove mineralocorticoid receptors mediation.

## Material and Methods

### Experimental model

The protocol of the study was approved by The Universidad Complutense Ethics Review Board and followed the current guidelines of the European Union and granted and approved by the Universidad Complutense Ethics Review Board following the National Guideline 53/2013. Rats were kept in a quiet room at constant temperature (20–22°C) and humidity (50%–60%) and fed standard rat chow and tap water *ad libitum*. Male Wistar rats 254±2 g; Harlam Iberica, Barcelona, Spain) were used in the study. Forty rats were divided in four groups (N = 10 per group): Aldosterone group (Aldo) which received an injection of aldosterone (1mg^-1^kg^-1^day; Sigma Aldrich) dissolved in corn oil and NaCl 1% in drinking water, spironolactone group (Spiro) which received an injection of spironolactone (200 mg^-1^kg^-1^ day; Sigma Aldrich), aldosterone+spironolactone group (Aldo+Spiro) which received an injection of aldosterone (1mg^-1^kg^-1^day; Sigma Aldrich) dissolved in corn oil and NaCl 1% in drinking water together with injection of spironolactone (200 mg^-1^kg^-1^ day; Sigma Aldrich) and, control group (Control) which received an injection of vehicle. The period of the study was 3 weeks. To measure systolic blood pressure (SBP) and diastolic blood pressure (DBP) blood pressure, at the end of the treatment period, the tail-cuff method was used [15]. After measuring hemodynamic parameters, the animals were killed, the kidney removed, weighed, and rapidly frozen in liquid nitrogen for molecular studies. Kidney weight to body weight ratio was used as the index of renal hypertrophy.

### Blood urine nitrogen (BUN) and creatinine levels

Serum BUN concentration was determined using a commercial kit (Pars Azmoon Co., Tehran, Iran), according to manufacturer’s instructions. Serum creatinine was measured by Jaffe’s method, using a Commercial Kit (Pars Azmoon Co., Tehran, Iran).

### RNA extraction and real-time PCR

Total RNA from renal tissues was isolated by Trizol (Invitrogen). cDNA was obtained by reverse transcription with the High Capacity cDNA Archive Kit (Applied Biosystems). Real-time PCR was performed on an ABI Prism 7500 PCR system (Applied Biosystems) using the DeltaDelta Ct method. Expression of genes of interest was reflected as ratios to Glyceraldehyde 3-phosphate dehydrogenase (GADPH). Pre-developed primers and probes assays: GADPH, arginase I (ArgI), interleukin 10 (IL-10), interferon gamma (IFN-γ), and inducible nitric oxide synthase (iNOS, Applied Biosystems).

### Western blot

The preparation of protein samples from renal tissues was performed as previously [16]. Antibodies to rabbit polyclonal anti-Collagen I (1:1000, AbDSerotec, Oxford, UK), anti- connective tissue growth factor (CTGF, 1:1000, Abcam, Cambridge, UK), anti-Fibronectin (FN 1:1000 Millipore, Germany), anti-TNF-α (1:1000, Abcam, Cambridge, UK), anti-matrix metalloproteinase 2 (MMP2, 1:1000, Abcam, Cambridge, UK), anti- cluster of differentiation 68 (CD68, 1:200, Abcam, Cambridge, UK), anti-arginase I (Arg-I, 1:1000 Santa Cruz, anti-serum and glucocorticoid kinase 1 (SGK-1, 1:1000, AbcamCambridge, UK) and monoclonal anti-α-tubulin antibody (1:10000, Sigma-Aldrich, Spain), were used.

### Renal collagen content

To asses renal collagen content, paraffin-embedded kidneys were cut into 4-mm slices and stained with Sirius Red F3BA (0.5% in saturated aqueous picric acid; Aldrich Chemical Company, Madrid, Spain). Four different sections of each slide of the kidney and ten photographs from each section were taken using an image analysis system (Leica Microsystems, Barcelona, Spain). A single investigator, blinded to the nature of the samples, performed the analyses.

### Flow cytometry and cell purification

Kidneys were decapsulated, minced and incubated with collagenase (0.5 mg/mL, Sigma-Aldrich) for 30 min at 37°C. After erythrocyte lysis, single-cell suspensions were prepared in staining buffer (2% FCS in Dulbecco´s PBS). Cells were stained with CD45-PE-Cy7 (BioLegend), CD86-PE (BioLegend) and CD163-APC (Bio-Rad). Then cells were washed, fixed, and permeabilized using the BD Cytofix/-Cytoperm kit (BD Bioscences) and subsequently incubated with CD68-FITC (Bio-Rad). Finally, the cells were stained using the Live/Dead Exclusion Fixable Violet Dead Cell Stain kit (Invitrogen, Carlsbad, CA). Cell suspensions were analysed in a FACSAria I (BD Biosciences) apparatus with the FlowJo (Tree Star) software packages. Rat peritoneal macrophages were isolated from the peritoneal cavity as previously described [17], and used as positive control for macrophage markers detection by flow cytometry.

### Clodronate administration

To study the effect of clodronate, an inhibitor of monocyte/macrophage influx, liposomal clodronate or control (PBS) liposomes (Clodronateliposomes, Amsterdam, The Nederlands), were administered intraperitoneally to the rats (20 mg/kg) on five separate days: day 0, 4, 9, 14, 18 of aldosterone (1mg^-1^kg^-1^day) + 1% NaCl treatment, according to previous publications [18,19]. Thus, the rats were randomly assigned to 4 different groups (n = 5/group): Control + PBS liposome, aldosterone+PBS liposome, control+clodronate liposome, or aldosterone+clodronate liposome.

### Statistical analysis

The data was analysed using a one-way analysis of variance, followed by a Newman-Keuls test if differences were noted (GraphPad Software Inc., USA). A p-value of 0.05 or less was considered significant.

## Results

### Blood pressure and renal function

Aldosterone+salt treated rats presented higher SBP and DBP levels than control rats (p<0.05). Treatment with the mineralocorticoid receptor antagonist spironolactone significantly reduced SBP and DBP (p<0.05). Finally, we observed elevated H_2_0 consumption and consequent higher urine excretion after the treatment of aldosterone. Spironolactone treatment in normal rats did not affect any of these parameters. Creatinine clearance and BUN were comparable in all groups (Table 1).

**Table 1:** Biological parameters in the different experimental groups.

	Control	Spiro	Aldo+Salt	Aldo+Salt+Spiro
SBP (mm Hg)	120±2.3	118±6.2	143±4.1*	115±2.1#
DBP (mm Hg)	85±3.2	80±1.1	105±6.4*	91±5.1#
Body weight (g)	304.3±18.2	298.2±15.4	311.5±6.8	294.5±10.2
KW/BW (mg/g)	0.32±0.03	0.32±0.05	0.37±0.03*	0.34±0.03
H_2_O Consumption (mL)	24.37±2.61	31.0±4.12	60.00±18.89*	39.29±8.86#
Urine (mL)	13.00±2.72	13.29±5.96	44.25±17.21*	26.25±12.66#
Creatinine (mg/dL)	30.69±1.5	28.5±2.8	31.3±2.6	30.4±3.6
BUN (mg/dL)	0.36±0.02	0.36±0.06	0.34±0.02	0.32±0.05

KW/BW, kidney weight/100 g body weight.

*p<0.05 versus Control

# p<0.05 versus Aldo+ Salt.

### Renal morphological alterations

**Fig 1:**
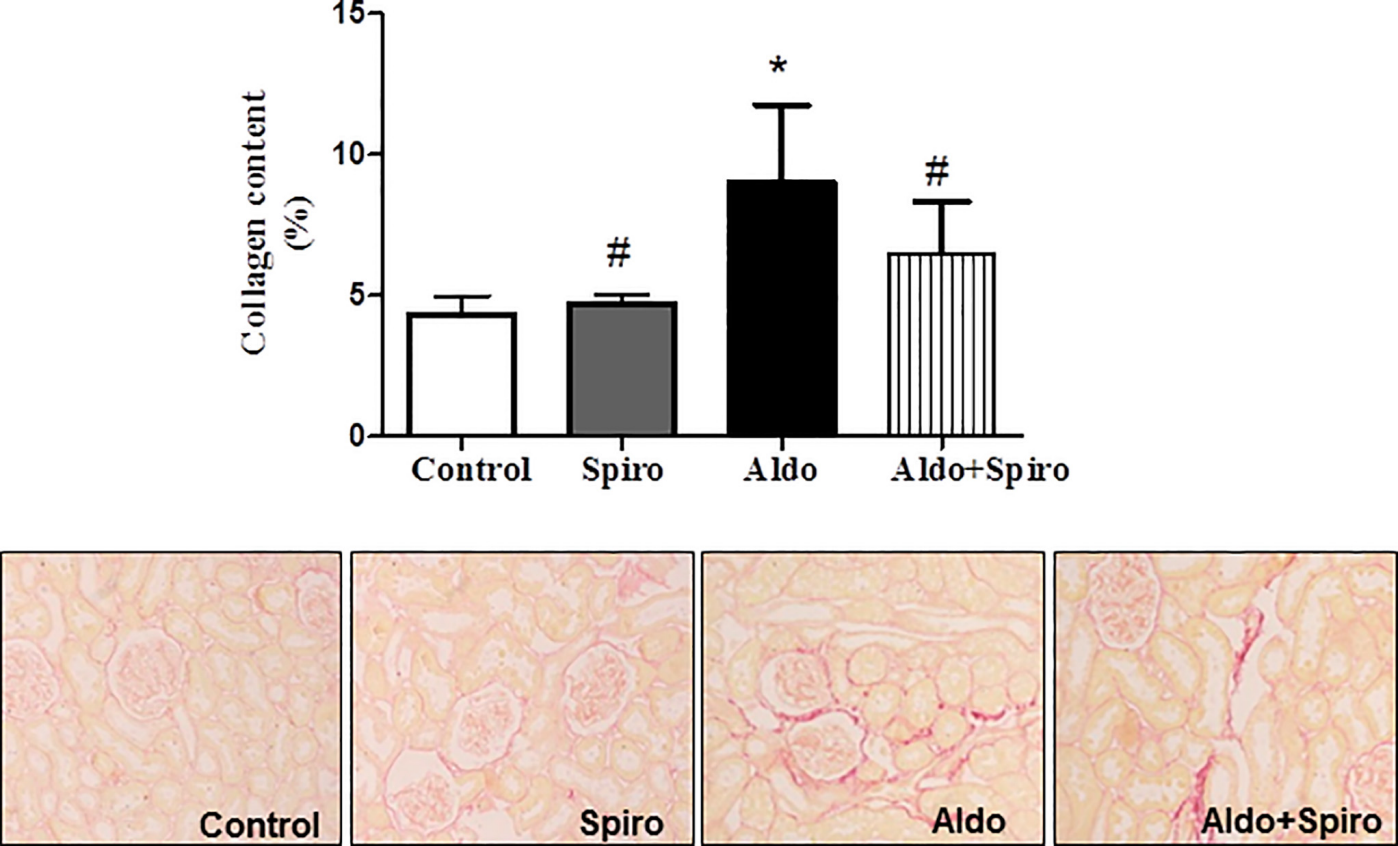
Kidney collagen content by Sirius red (A) and Masson trichrome and representative images of collagen content by Hematoxilin/Eosin (B) in control (CONTROL), spironolactone treated animals (SPIRO), aldosterone+salt-treated animals (ALDO) and aldosterone+salt plus spironolactone treated animals (ALDO+SPIRO). Data are expressed as mean ± SEM. *p<0.05 vs. CONTROL; ^#^p<0.05 vs. ALDO

Masson trichrome, Sirius red and Hematoxilin/Eosin staining were used to examine the morphological changes in the kidneys (Fig 1A and 1B). The kidneys from control animals showed normal structure. However, Aldosterone treatment increased extracellular matrix deposition, as determined by Sirius red (p<0.05). Fibrosis was mainly interstitial and perivascular. Treatment with spironolactone decreased extracellular matrix deposition (p<0.05).

### Renal hypertrophy and fibrosis

**Fig 2:**
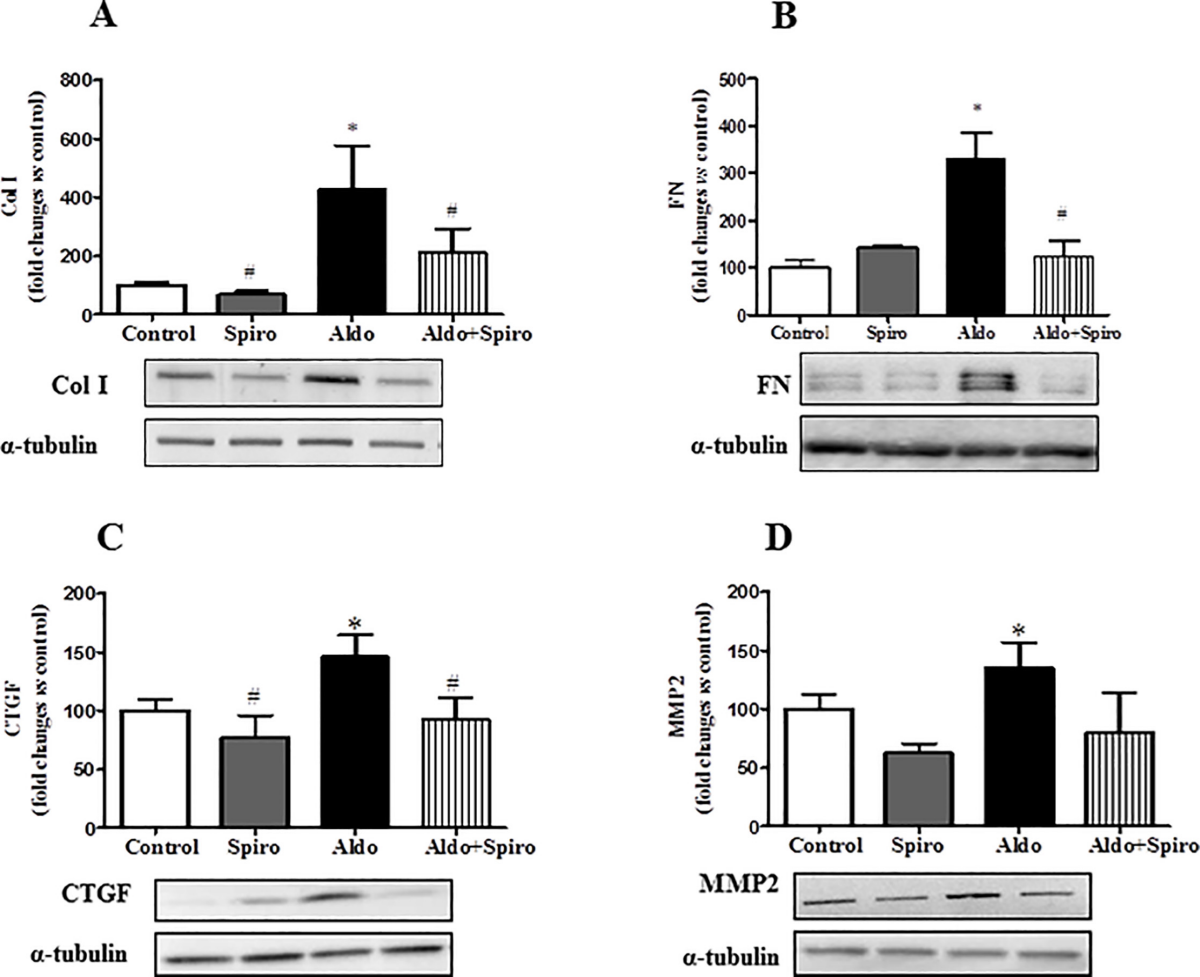
Quantitative analyses of protein levels measured by Western blot for ColI (A), FN (B), CTGF (C) and MMP-2(D) in control (CONTROL), spironolactone treated animals (SPIRO), aldosterone+salt-treated animals (ALDO) and aldosterone+salt plus spironolactone treated animals (ALDO+SPIRO). Data are expressed as mean ± SEM. *p<0.05 vs. CONTROL; ^#^p<0.05 vs. ALDO

At the end of the study, all groups presented comparable body weight, however the relative kidney weight (KW/BW) was higher (p<0.05) in aldosterone + salt-treated rats than in controls (Table 1). In order to determine the molecular mechanism involved in aldosterone-mediated fibrosis we performed western blot in kidney homogenates. Western-blot analysis revealed that Col I and FN were the main component of the fibrotic extracellular matrix in aldosterone + salt treated-animals **(**Fig 2A and 2B). Increased fibrotic mediators, such as connective tissue growth factor (CTGF), was observed in aldosterone + salt treated-animals (Fig 2C). MMP2 protein levels were higher (p<0.05) in aldosterone + salt–treated rats compared with controls. All these parameters were reduced (p<0.05) by spironolactone treatment. Spironolactone did not affect any of these parameters in aldosterone + salt–untreated rats (Fig 2D).

### Inflammation and macrophage phenotypes

**Fig 3:**
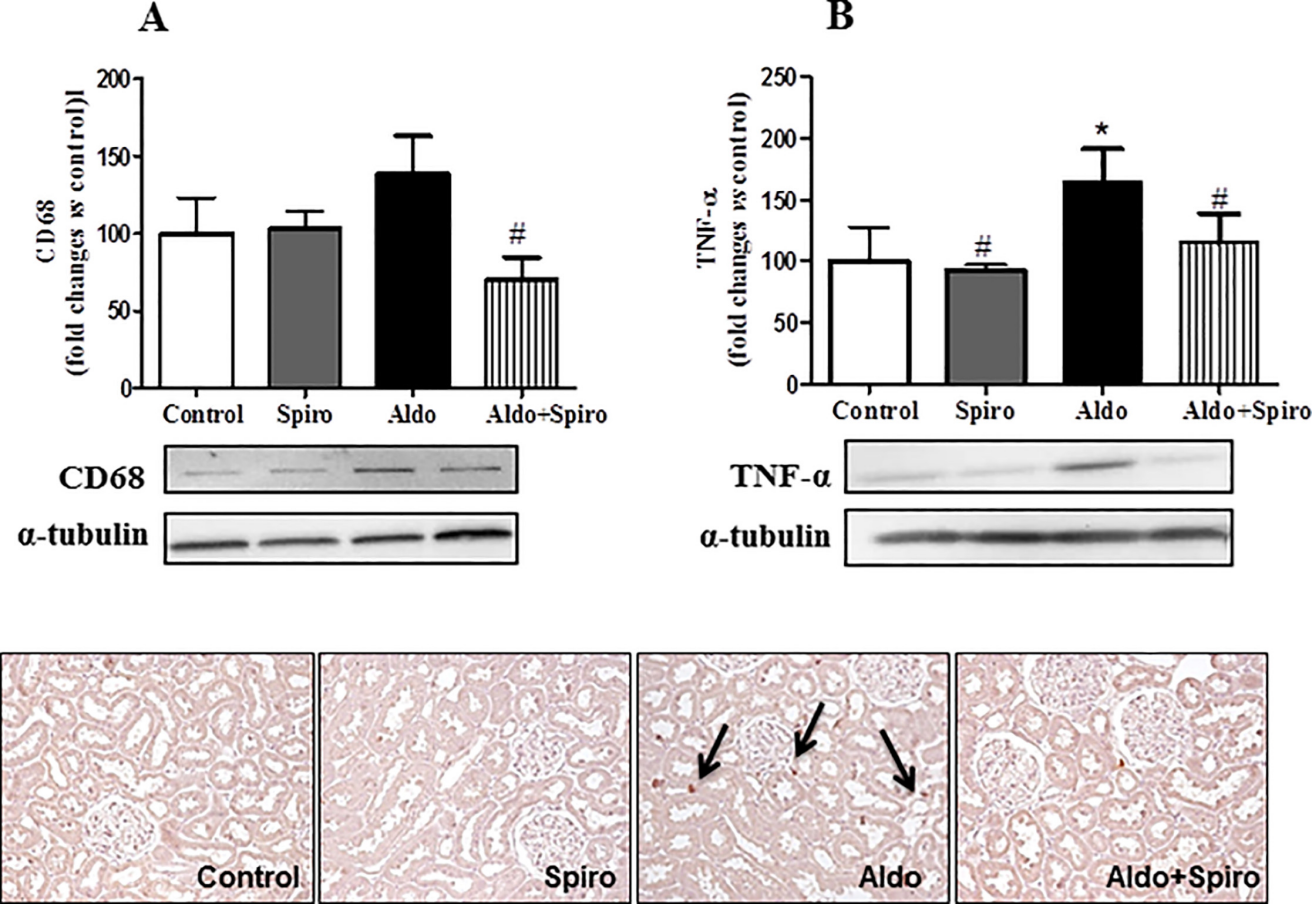
Quantitative analyses of protein levels measured by Western blot for CD-68 (A), TNF-α (B) and representative images of CD68 staining (C) in control (CONTROL), spironolactone treated animals (SPIRO), aldosterone+salt-treated animals (ALDO) and aldosterone+salt plus spironolactone treated animals (ALDO+SPIRO). Data are expressed as mean ± SEM. *p<0.05 vs. CONTROL; ^#^p<0.05 vs. ALDO

**Fig 4:**
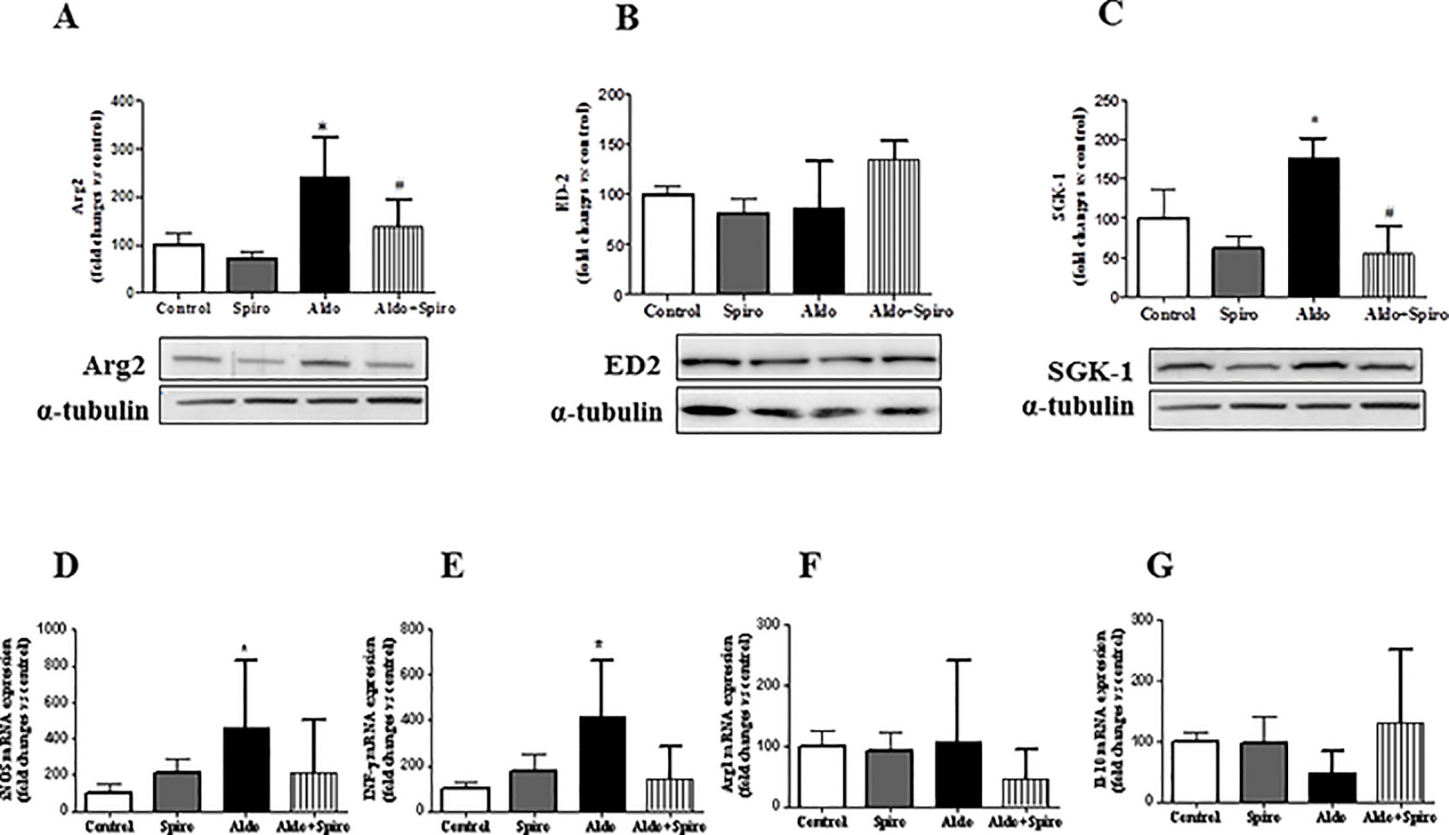
Quantitative analyses of protein levels measured by Western blot for Arg2 (A), ED2 (B) and SGK-1 (C) and mRNA levels measured by RT-PCR for iNOS (D), INFγ (E), Arg1 (F) and Il-10 (G) in control (CONTROL), spironolactone treated animals (SPIRO), aldosterone+salt-treated animals (ALDO) and aldosterone+salt plus spironolactone treated animals (ALDO+SPIRO). Data are expressed as mean ± SEM. *p<0.05 vs. CONTROL; #p<0.05 vs. ALDO.

An increased inflammation, characterized by increased CD68 and TNF-α expression was observed in aldosterone + salt treated rats, as compared with control group(p<0.05) (Fig 3A and 3B). These inflammatory parameters were reduced (p<0.05) by spironolactone treatment. SGK-1 is one of the main mediator of aldosterone actions and it was highly increased in aldosterone+salt treated rats (p<0.05). However, this expression was markedly reduced by treatment with spironolactone (Fig 4A). We then determined macrophage phenotype markers to characterize renal M1/M2 macrophage distribution. Increased Arg2 protein expression, a M1 macrophage marker, was observed in kidney from aldosterone treated rats, whereas no significant differences were observed in ED2 protein content (Fig 4B and 4C). To confirm these results we determined mRNA expression of different M1 markers such as IFN-γ and iNOS. As reported in Fig 4D and 4E, an increased mRNA expression of iNOS and IFN-γ was observed after aldosterone + salt treatment. All these parameters, but iNOS which presented a tendency to decrease, were reduced (P < 0.05) by spironolactone treatment. Arg1 or IL-10 mRNA expression (M2 macrophage markers) did not change after aldosterone administration (Fig 4F and 4G). All together our results show a polarization towards a M1 phenotype by aldosterone that may be partially reduced after MR blockade.

**Fig 5:**
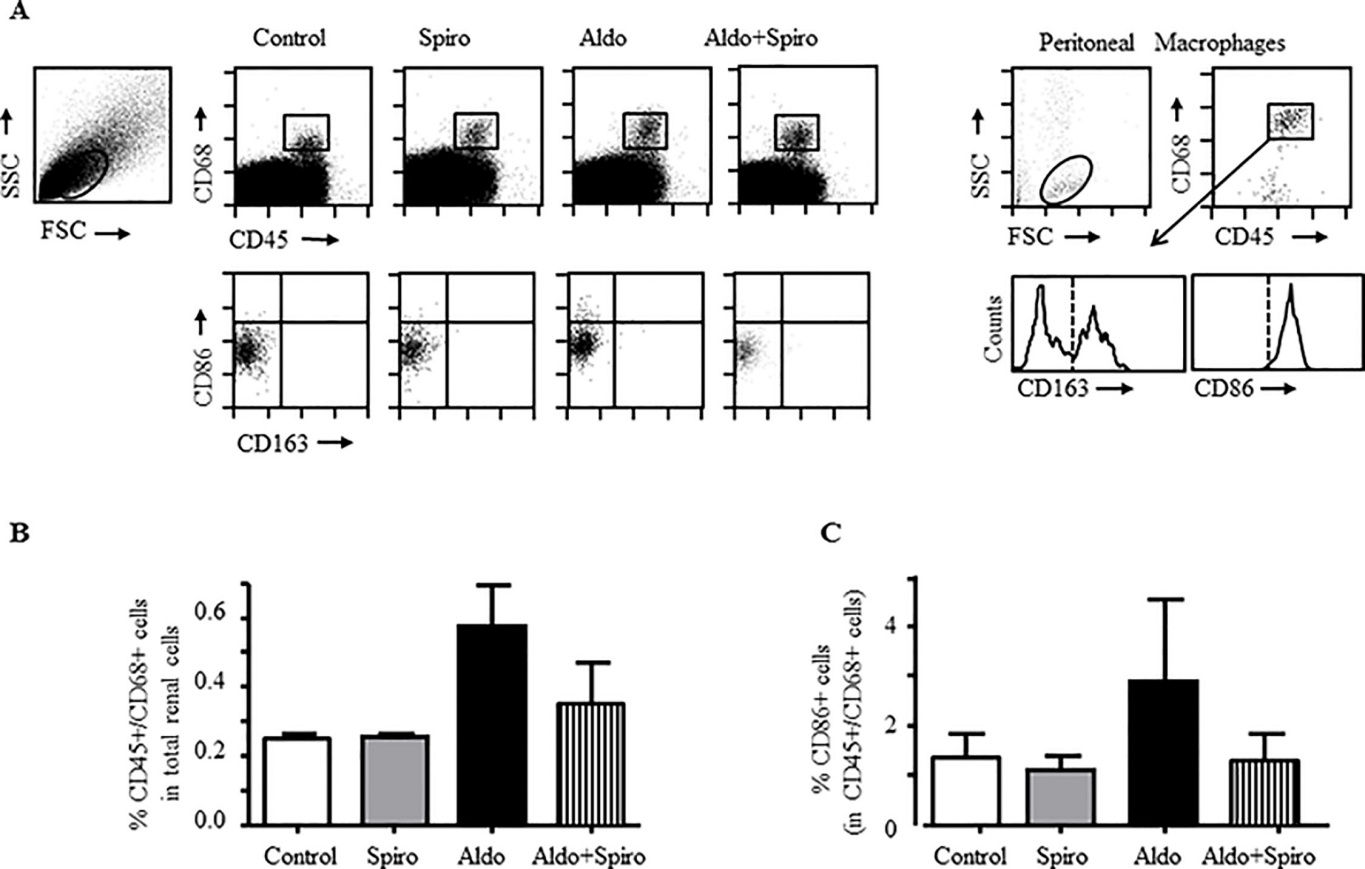
Inflammatory and anti-inflammatory macrophage markers by flow cytometry in kidneys from aldosterone treated rats. (A) Representative dot-plots of the CD45 and CD68 staining in cells gated in the low SSC/FSC window (A, left) from kidney suspensions in control (Control), spironolactone treated animals (Spiro), aldosterone+salt-treated animals (Aldo) and aldolsterone+salt plus spironolactone treated animals (Aldo+Spiro). The box inside the dot plots identify cell population expressing CD45 and CD68. Cells included in this box are analysed in down dot plots for CD86 and CD163 expression. Dot plot for peritoneal macrophages as positive control for the detection of CD45, CD68 (A, right). The histogram represent the CD163 expression in CD45^+^/CD68^+^ cells. The vertical dotted line show the isotype control. (B) The bar chart represents the relative number of CD68^+^ cells in kidneys from control (Control), spironolactone treated animals (Spiro), aldosterone+salt-treated animals (Aldo) and aldolsterone+salt plus spironolactone treated animals (Aldo+Spiro). The data are the means ± SEM, n = 2. (C) Graph represents the relative number of CD86 positive cells on CD68 population in control (Control), spironolactone treated animals (Spiro), aldosterone+salt-treated animals (Aldo) and aldolsterone+salt plus spironolactone treated animals (Aldo+Spiro).The data are the means ± SEM, n = 2.

In a further step, macrophages were isolated from treated kidneys and the expression of M1/M2 markers was quantified by flow cytometry. We first discriminated macrophages populations in kidneys according to the dual presence of CD45 (leukocyte common antigen) and CD68 (general macrophage marker) and then analyzed the expression of CD86 (M1 marker) and CD163 (M2 marker), as previously reported [20,21]. Analysis of renal cells isolated from kidneys tissues showed an increase in CD45^+^/CD68^+^ macrophages in aldosterone-treated rats (Fig 5A and 5B). Moreover, expression of the M1 marker CD86 was only increased in CD45^+^/CD68^+^ macrophages from aldosterone-treated rats (Fig 5C); whereas no CD163 expression was detected in CD45^+^/CD68^+^ macrophages from any group (Fig 5A). A partial reduction of CD86 expression was observed after MR blockade in aldosterone-treated rats. Macrophages isolated from rat peritoneum were used as positive control for the detection of CD86 and CD163 in CD45^+^/CD68^+^ cells (Fig 5A).

### Clodronate administration reduced renal macrophage infiltrate and inflammatory markers, but not fibrosis, in aldosterone-treated rats

**Fig 6:**
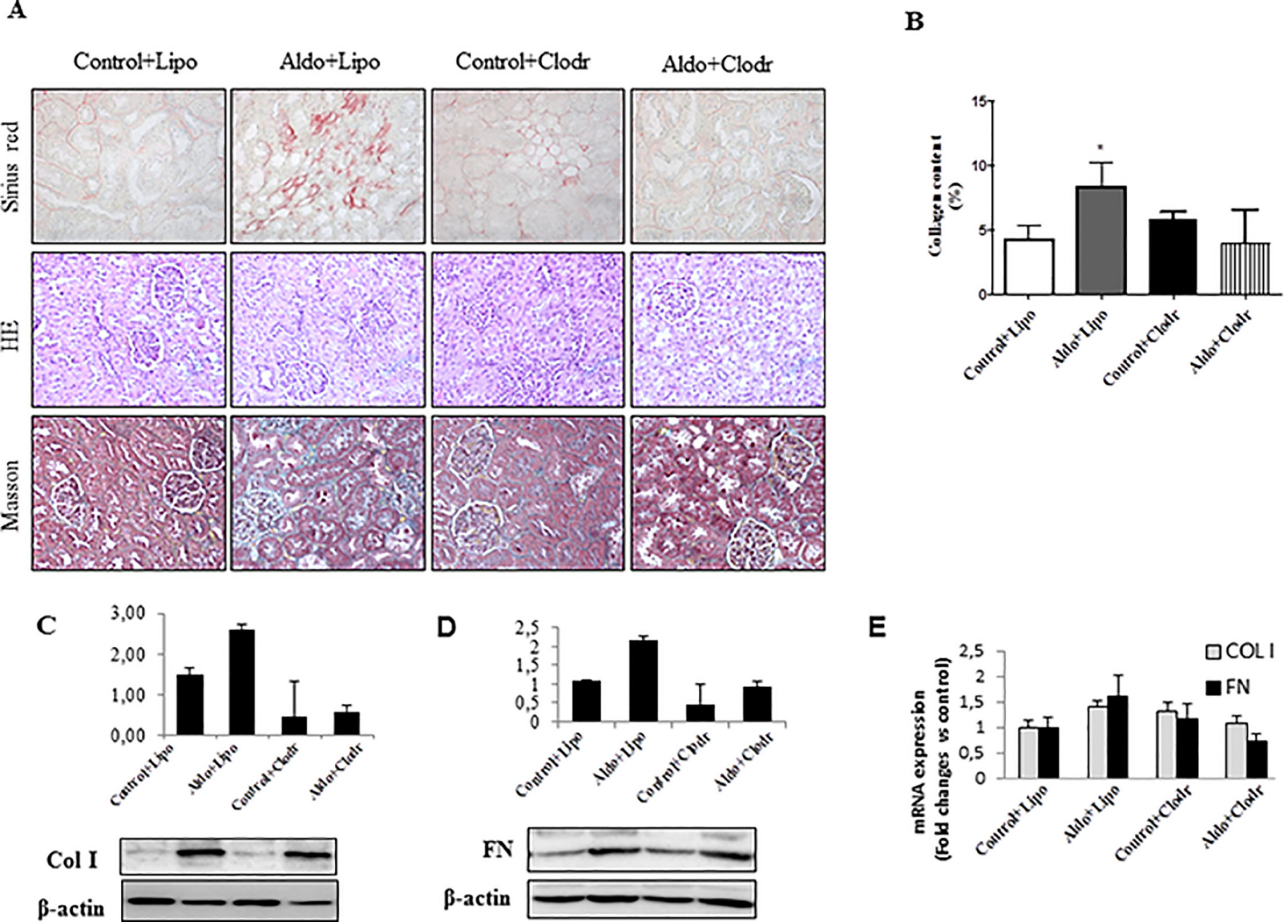
(A) Representative images showing Sirius red staining, Masson trichrome and Hematoxilin/Eosin staining in control+PBS liposome (Control+Lipo), aldosterone+PBS liposome (Aldo+Lipo), control+clodronate liposome (Control+Clodr), or aldosterone+clodronate liposome (Aldo+Clodr) treated animals. Kidney collagen content (B), quantitative analyses of Col I and Fibronectin (FN) protein (C-D) and mRNA expression (E) in treated rats. Data are expressed as mean ± SEM. *p<0.05 vs Control+Lipo; #p<0.05 vs. Aldo+Lipo.

**Fig 7:**
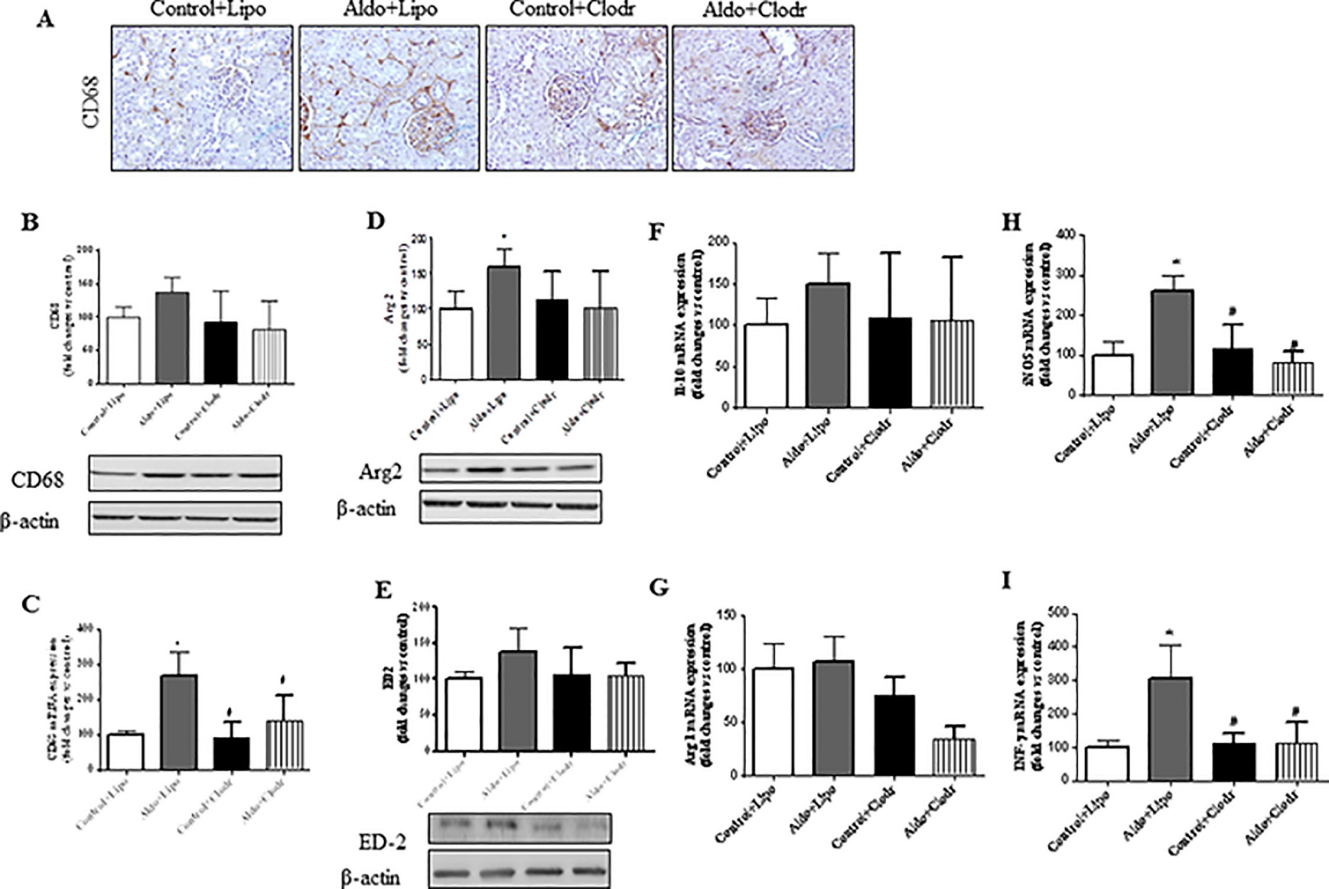
(A) Representative images showing CD68 staining in control+PBS liposome (Control+Lipo), aldosterone+PBS liposome (Aldo+Lipo), control+clodronate liposome (Control+Clodr), or aldosterone+clodronate liposome (Aldo+Clodr) treated animals. Quantitative analyses of CD68 protein (B) and mRNA (C) levels in treated rats. Quantitative analyses of Arg2 (D) and ED-2 (E) protein levels and IL-10 (F), Arg 1 (G) iNOS (H) and INFγ (I) mRNA expression in treated rats. Data are expressed as mean ± SEM. *p<0.05 vs Control+Lipo; #p<0.05 vs. Aldo+Lipo.

To determine whether macrophages were directly implicated in aldosterone-mediated fibrotic effects, we depleted macrophages by using clodronate liposomes. Neither clodronate- or control-liposomes had an effect on clinical parameters or kidney structure in control rats (S1 Table and Fig 6). Administration of liposomal clodronate in aldosterone-treated rats reduced significantly systolic blood pressure (p<0.05), ameliorated renal morphology and partly reduced H_2_O consumption, urine excretion, the relative kidney weight (KW/BW), and extracellular matrix deposition (sirius red) as well as profibrotic mediators, such as Col I, FN and CTGF (Fig 6 and S1 Fig), as compared with aldosterone-treated rats that received control liposomes. Kidneys from aldosterone-treated rats showed a significant decrease in both macrophage number and CD68 mRNA expression upon clodronate administration (Fig 7A–7C). Consistent with our previous data (Fig 4), this treatment also reduced Arg2 at the protein level and the mRNA expression of INF-γ or iNOS (Fig 7D, 7F and 7H), all of them M1 markers; whereas non-significant reduction was observed in M2 markers (IL-10, Arg1 and ED2) (Fig 7F and 7G). Thus, despite effective reduction of macrophage influx and renal inflammatory response after clodronate treatment, interstitial fibrosis was not fully prevented by this intervention.

## Discussion

The present study shows that administration of aldosterone plus salt in rats promotes a polarization towards a M1 phenotype by aldosterone, accompanied by hypertension, renal damage, hypertrophy and fibrosis. All these effects were blocked by MR antagonism with spironolactone, supporting the protective effects of MR blockade in hypertensive renal disease. Moreover, we observed a reduction in aldosterone-mediated renal inflammation (macrophages and mediators of inflammation) after liposomal clodronate treatment, whereas interstitial fibrosis was only partially reduced after this intervention.

It is well known that aldosterone is related to development of inflammation and fibrosis in kidney [2,9]. Our study shows a pro-inflammatory action of aldosterone in the kidney by an increment in both TNF-α protein expression and IFN-γ mRNA expression level which decreased when spironolactone was administered. As previously described, the mechanistic processes induced by aldosterone include dependent MR activation and a close link with the interplay with Ang II levels together with sodium and potassium ionic transport [22,23].

Although inflammation may promote fibrosis; aldosterone can also directly induce the expression of pro-fibrotic molecules. In the present study, aldosterone plus salt treatment increased relative kidney weight and collagen content. Furthermore, aldosterone stimulated CTGF expression in the rat kidney, which could mediate collagen production through MR activation, as suggested by the reduction caused by spironolactone treatment. CTGF is a key mediator of matrix protein formation, and upregulated in several fibrotic renal diseases, including diabetic nephropathy and glomerulosclerosis [24–26]. Our results showed elevation of MMP2 in aldosterone treated rats compared to controls which justify the observed reduced collagen degradation and enhancement of kidney fibrosis. *In vitro* studies in rat mesangial cells as well as in renal fibroblasts have shown an extracellular degradation due to aldosterone deleterious effects [27].

Protein expression of SGK1, one of the key mediators of aldosterone functions, was increased in the kidney of the aldosterone-treated rats and normalized after treatment with spironolactone. It has been reported that aldosterone induces phosphorylation of SGK-1 in a MR-dependent manner [8,28]. SGK-1 would be enhancing sodium intake as it has been reported in human renal proximal tubule cells, where aldosterone-stimulated phosphorylation of SGK1 corresponded to the increase of sodium transporter expression [29]. The increased salt retention plays a central role in the development of hypertension and renal fibrosis leading to maladaptive conditions like salt-sensitive hypertension and chronic kidney disease [30].

Renal protection by spironolactone was accompanied by a significant decrease in blood pressure and this reduction may contribute to the attenuation of renal damage and inflammation. As previously mentioned, sodium handling by the kidney is a major determinant of blood pressure changes and is controlled by physiological mechanisms including the sympathetic nervous system, hormones and inflammatory mediators [31]. In humans, increased blood pressure has been correlated with progression of nephropathy and incident end-stage renal disease in the general population. Indeed, it has been proposed that hypertension is related to more than 80% of patients with chronic kidney disease contributing to more advance chronic kidney disease as well as cardiovascular events [32]. Taking into account the evidences, it seems quite relevant the effort to lower blood pressure in order to reduce progression of chronic kidney disease [33].

The macrophage plays a key role in cardiovascular inflammation, fibrosis and remodeling induced by aldosterone and high salt intake [34]. While macrophage infiltration has been reported in aldosterone plus salt treated rats[9], the role or phenotype of the macrophages in this experimental model remains unknown. Here, we identified that aldosterone + salt administration mediates inflammatory M1 macrophage phenotype *in vivo*. Macrophages are heterogeneous cells that play different functions [35,36]. M1 macrophages release pro-inflammatory chemokines and promote fibrosis; whereas M2 macrophages are associated with immunoregulatory and tissue-remodeling functions [37]. Increased renal infiltration of pro-inflammatory M1 macrophages have been described in lupus nephritis [38] and non-immune renal diseases [39]. However, to our knowledge, this is the first study reporting increased M1-macrophage infiltration in the aldosterone plus salt experimental model of hypertension. A number of *in vitro* studies have shown that macrophage phenotype changes in response to different stimuli, including aldosterone [11]. Indeed, aldosterone promoted M1 polarization and production of pro-inflammatory cytokines (TNF-α, CCL2 and CCL5) and pro-fibrotic proteins (TGF-β and PAI-1) in macrophages in culture [13,40]. In agreement with this observation, our study showed thatM1 macrophage markers (Arg2 and iNOS) were associated with an augmented TNF-α content in the kidney from aldosterone-treated rats. We also observed that aldosterone-mediated pro-inflammatory response was prevented by MR antagonism with spironolactone. MR is expressed in infiltrating macrophages and recent evidences indicate a role for MR in macrophage polarization [41]. Thus, rat peritoneal macrophages treated with aldosterone resulted in increased expression of the M1 classical activation markers TNF-α, which was blocked by the MR antagonist spironolactone [40]. Similarly, in an immortalized mouse microglial cell line, which is macrophage-like cells of the central nervous system, MR activation with aldosterone potentiated LPS-induction of the pro-inflammatory cytokines TNFα and IL-6 in an MR dependent way [42]. Studies conducted in peritoneal macrophages taken from mice with MR specifically deleted from macrophages (Mac-MR-KO) reported that MR-deficient macrophages showed reduced expression of M1 markers, decreased responsiveness to LPS-induced activation, and a shift toward the alternative-activated M2 phenotype [12]. Overall, the available data support the conclusion that the MR in macrophages contributes to classical macrophage activation to the M1 pro-inflammatory phenotype, and that MR blockade or deletion in macrophages prevents classical macrophage activation, which is line with the results by our study.

Since classical (M1) and alternative (M2) activation have been reported to be competing pathways, we also determined the effect of aldosterone administration on M2markers. However, no significant differences on M2 markers were observed, suggesting that aldosterone does not promote macrophage differentiation toward alternative macrophage differentiation *in vivo*. Several studies support the notion that M2 macrophages may promote the development of fibrotic lesions. These alternatively activated macrophages synthesize a number of pro-fibrotic factors, such as TGF-β [43] and CTGF [44]. In addition, incubation of fibroblasts with alternatively activated macrophages promotes fibroblast proliferation and collagen synthesis [45,46]. Since no increased presence of M2 macrophage markers was observed in our study after aldosterone administration, our data suggest that this macrophage subtype not contributed to renal fibrosis in this hypertensive experimental model of renal damage.

In order to investigate the specific role of macrophages in this hypertensive experimental model, we depleted macrophages using clodronate liposomes. Administration of clodronate in aldosterone-treated rats prevented the increase in tissue macrophage number and also reduced some inflammatory markers at the mRNA level, but did not show major changes neither on the clinical parameters nor interstitial fibrosis. Therefore, this study shows that aldosterone-induced fibrosis cannot be prevented by reducing macrophage influx. We could hypothesize that, in this experimental model, other resident renal cells, such as tubular epithelial cells or fibroblasts may be continuously activated by aldosterone, being the main source of components of the extracellular matrix and therefore promoting fibrosis [6].

In conclusion, administration of aldosterone in presence of salt enhance M1 macrophage infiltration in the kidney. These aldosterone-mediated effects together with the aldosterone mediator, SGK-1; are effectively attenuated by the selective MR antagonist spironolactone, supporting the protective effects of MR blockade in hypertension-driven renal injury.

## Supporting Information

S1 Fig Quantitative analyses of protein levels measured by Western blot for CTGF in control+PBS liposome (Control+Lipo), aldosterone+PBS liposome (Aldo+Lipo), control+clodronate liposome (Control+Clodr), or aldosterone+clodronate liposome (Aldo+Clodr) treated animals. (TIF)

S1 Table Biological parameters in the different experimental groups. KW/BW, kidney weight/100 g body weight.*p<0.05 versus Control; # p<0.05 versus Aldo+Lipo.
(DOCX)
