# Supplementary figures and images for: Aldosterone Induces Renal Fibrosis and Inflammatory M1-Macrophage Subtype via Mineralocorticoid Receptor in Rats
Source: PLoS One. 2016 Jan 5;11(1):e0145946. doi: 10.1371/journal.pone.0145946 (PMC4701403; doi:10.1371/journal.pone.0145946)

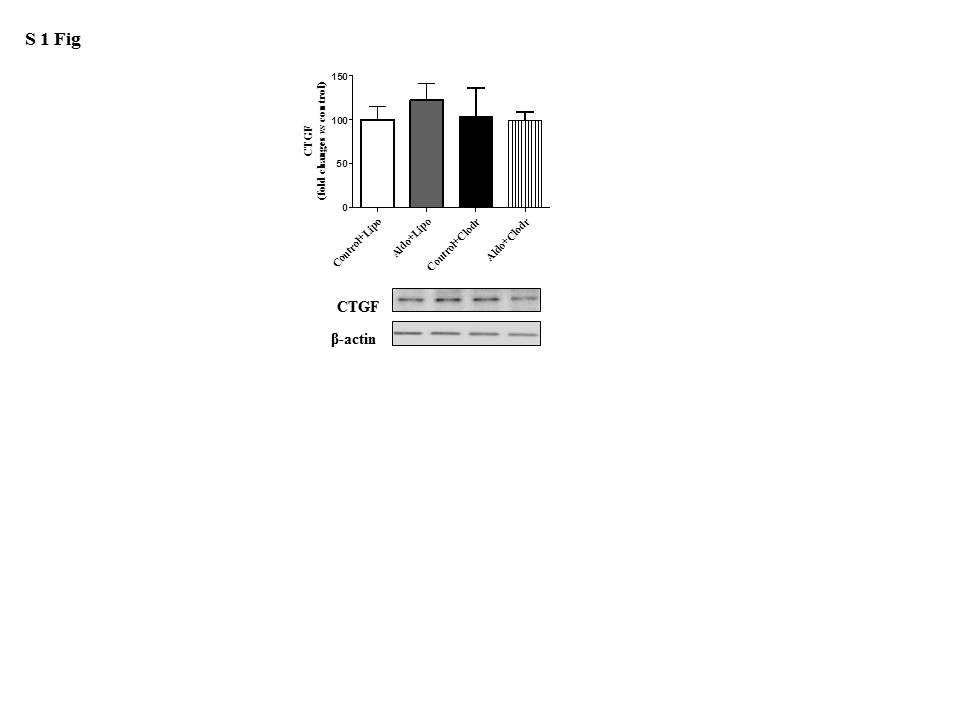

Supplement: S1 Fig — (TIF) [file pone.0145946.s001.tif]
