# Supplementary material for: Aldosterone Induces Renal Fibrosis and Inflammatory M1-Macrophage Subtype via Mineralocorticoid Receptor in Rats
Source: PLoS One. 2016 Jan 5;11(1):e0145946. doi: 10.1371/journal.pone.0145946 (PMC4701403; doi:10.1371/journal.pone.0145946)
Supplement: S1 Table — KW/BW, kidney weight/100 g body weight.*p<0.05 versus Control; # p<0.05 versus Aldo+Lipo. (DOCX) [file pone.0145946.s002.docx]

|  | **Control+Lip** | **Aldo+Lip** | **Control+Clodr** | **Aldo+Clodr** |
| --- | --- | --- | --- | --- |
| SBP (mmHg) | 111±8 | 137±2* | 114±5^#^ | 112±5^#^ |
| DBP (mmHg) | 86±7 | 112±2* | 94±1^#^ | 98±1 |
| Body weight (g) | 305±23 | 308±10 | 351±18 | 303±12 |
| KW/BW (mg/g) | 0,31±0.006 | 0.41±0.026* | 0.29±0.020^#^ | 0.36±0.012 |
| H_2_O Consumption (mL) | 31±7 | 62±10* | 26±5^#^ | 40±5 |
| Urine (mL) | 18±3 | 41±5* | 11±1^#^ | 28±6 |

**Supplemental Table 1.** Biological parameters in the different experimental groups.
